# Changes in fatty acids composition, antioxidant potential and induction period of UHT-treated tea whitener, milk and dairy drink

**DOI:** 10.1186/s12944-019-1161-x

**Published:** 2019-12-07

**Authors:** Muhammad Ajmal, Muhammad Nadeem, Muhammad Imran, Zarina Mushtaq, Muhammad Haseeb Ahmad, Muhammad Tayyab, Muhammad Kamran Khan, Nabila Gulzar

**Affiliations:** 1 Department of Dairy Technology, University of Veterinary and Animal Sciences, Lahore, Pakistan; 2 Institute of Home and Food Sciences, Faculty of Life Sciences, Government College University, Faisalabad, Pakistan; 3 Institute of Biochemistry and Biotechnology, University of Veterinary and Animal Sciences, Lahore, Pakistan

**Keywords:** Tea whitener, UHT-treated milk, Dairy drink, Antioxidant capacity, Fatty acid profile, Induction period, Rancimat

## Abstract

**Background:**

In developing and developed countries, several versions of safe and shelf-stable Ultra High Temperature, UHT-treated products are manufactured. Terminologies and formulations of UHT-treated tea whitener, milk and dairy drink considerably vary. Comprehensive studies have been performed on UHT-treated milk; however, fatty acids compositional changes and oxidation status of UHT-treated tea whitener and dairy drink at different storage intervals have not been reported in literature.

**Methods:**

UHT-treated tea whitener, milk and dairy drink samples (450 each) of the same manufacturing date were purchased from the market and stored at ambient temperature (25-30 °C) for 90 days. At the time of collection, all the samples were only one week old. Samples of UHT-treated tea whitener, milk and dairy drink were regarded as treatments and every treatment was replicated five times. Chemical composition, fatty acid profile, 2, 2-Diphenyl-1-picrylhydrazyle (DPPH) radical scavenging activity, total antioxidant activity, reducing power, antioxidant activity in linoleic acid system and induction period were determined at 0, 45 and 90 days of storage.

**Results:**

Fat content in freshly collected samples of UHT treated-tea whitener, milk and dairy drink were 6 and 3.5%. UHT treated milk had highest total antioxidant capacity, antioxidant activity in linoleic acid and 2, 2-Diphenyl-1-picrylhydrazyle (DPPH) free radical scavenging activity followed by UHT tea whitener and dairy drink. In freshly collected samples of UHT-treated milk, concentrations vitamin A and E were 0.46 μg/100 g and 0.63 mg/100 g, respectively. UHT-treated tea whitener had the lowest concentrations of vitamin A and E. With the progression of storage period, amount of vitamin A and E decreased. In freshly collected samples, amount of short, medium and unsaturated fatty acids in UHT-treated milk were 10.54, 59.71 and 27.44%, respectively. After 45 days of storage of UHT-treated milk, the loss of short, medium and unsaturated fatty acid was 7%, 7.1 and 5.8%, respectively. After 90 days of storage of UHT-treated milk, the loss of short, medium and unsaturated fatty acid was 8.53, 13.51 and 11.88%, accordingly. After 45 days of storage of UHT-treated tea whitener, the loss of medium and unsaturated fatty acid was 1.6 and 0.99%, respectively. After 90 days of storage, the loss of medium and unsaturated fatty acids were 8.2 and 6.6%, respectively. The induction period of fresh UHT-treated tea whitener, milk and dairy drink was 15.67, .74 and 7.27 h. Strong correlations were recorded between induction period and peroxide value of UHT-treated products.

**Conclusion:**

This investigation disclosed that UHT-treated tea whitener had 6% fat content with no short-chain fatty acids. Antioxidant capacity of UHT-treated milk was higher than dairy drink and tea whitener. Due to the presence of partially hydrogenated fat, oxidative stability of UHT-treated tea whitener was better than UHT-treated milk and dairy drink. Vitamin A and E was not found in UHT-treated tea whitener. For the anticipation of oxidative stability of UHT-treated milk, dairy drink and tea whitener, induction period/ Rancimat method can be used.

## Background

Milk and dairy products are main source of human nutrition [1]. Thermal processes are applied for the long-term preservation of fluid milk and dairy products. Thermal processing is performed to reduce/ eliminate the bacteria, limit enzyme activity and enhance the shelf life [2]. The effectiveness of heat treatment depends upon the heating method employed and time-temperature combination. UHT treatment is commercially applied to manufacture long life milk [3]. Ultra High Temperature (UHT) treatment is used for the manufacturing of commercially sterile fluid milk, this involves heating the milk to 135–150 °C followed by aseptic packaging [4]. UHT treated products can be stored for 3–6 months without refrigeration [5]. Heating of milk to UHT temperature consequences in declining the sensory attributes, nutritional value and oxidation of lipids [6]. Developing countries do not have well-established cold chain facilities, therefore, UHT milk is the most common source of milk in these areas. Depriving food sources and ever-increasing population of developing countries have led to the commercial scale production of tea whiteners and dairy drinks. Tea-whiteners are usually formulated from skim milk powder, partially hydrogenated palm oil (6%), stabilizer and emulsifier followed by high pressure homogenization (< 200 Bar), UHT treatment and aseptic packaging. The word UHT dairy drink is locally used to describe a product that is formulated from demineralized whey powder, anhydrous milk fat, stabilizer and emulsifier, upstream homogenization, UHT treatment and aseptic packaging [7]. Several studies have been performed on the characterization of UHT-treated milk, detailed investigation addressing the lipid oxidation and antioxidant features of tea whitener and dairy drinks was not reported in literature. Oxidation of lipids leads to the loss of nutritional value and the development of off-flavors during the storage of UHT-treated milk [8]. Milk fat contains about 25–28% unsaturated fatty acids, hence it is liable to oxidation, storage temperature, light, metals and enzymes may lead to oxidative and hydrolytic rancidity in UHT-treated milk during the storage [9]. Sunds et al. [10] studied the shelf life of UHT-treated milk in accelerated storage conditions however, transition in antioxidant properties of milk was not reported. Former studies have shown that, the development of off-flavors is the consequence of an imbalance between the concentration of antioxidants and pro-oxidants [11]. Production of several reactive oxygen species in aerobic creatures is a normal phenomenon e.g. superoxide, hydroxyl, peroxyl and alkoxy radicals [12]. When concentration of free radicals exceeds the defense system, state of oxidative stress occurs. Free radicals may cause carcinogenesis, cardiovascular diseases, necrosis and atherosclerosis [13]. In body and food systems, activities of free radicals may be minimized by the antioxidants. Milk naturally contains two distinct antioxidant defense systems and these may be broadly classified in two categories as fat soluble and water-soluble antioxidant systems [14]. Fat soluble antioxidant system is comprehended of vitamin A, E, and carotenoids. While, water solvable antioxidant defense mechanism is made up of casein, whey proteins, vitamin C, cysteine, valine, lactase, glutathione peroxidase and superoxide dismutase, zinc and selenium [15, 16]. Estimation of shelf life/oxidative stability of UHT-treated milk is a time taking phenomenon and sample of milk has to be stored for six months. Accelerated oxidation is widely used for the estimation of shelf life of fats, oils, cookies, potato chips and several foods, however, accelerated oxidations conditions are not formerly used for the anticipation of keeping quality of UHT-treated milk and dairy products. Former studies of Nadeem et al. [17] showed that accelerated oxidation can be used for the assessment of shelf life of milk fat. Khan et al. [18] reported that heating method, storage period and spoilage agent of UHT products are different from pasteurized milk as UHT products are commercially sterile and aseptically packaged with no live organism. Detailed investigation on the oxidative variations in UHT-treated tea-whitener, milk and dairy drink is required. This study aimed to examine the antioxidant capacity of UHT-treated tea-whitener, milk and dairy drink for a period of 90 days the basis of antioxidant assays and fatty acid profile.

## Methods

### Materials and experimental plan

UHT-treated tea whitener, milk and dairy drink samples (450 each) of the same manufacturing date were purchased from the market and stored at ambient temperature (25-30 °C) for 90 days. At the time of collection, all the samples were only week old. Samples of UHT-treated tea whitener, milk and dairy drink were regarded as treatments and every treatment was replicated five times.

### Chemical composition of milk

Chemical composition of UHT-treated tea whitener, milk and dairy drinks was determined by wet chemistry.

### Antioxidant assays

#### 2, 2-Diphenyl-1-picrylhydrazyle (DPPH) radical scavenging activity

DPPH free radicals scavenging activity in UHT-treated tea whitener, milk and dairy drink samples were determined by technique prescribed by Ye et al. [19]. The sample was prepared in test tube by mixing the 200 μL of milk sample in 1 mL of 100 μM DPPH solution. Spectrophotometer was used for the measurement of absorption at 517 nm.

#### Total antioxidant activity (TAC)

Sample (0.3 g) was measured in test tube and mixed with 3 ml each H_2_SO_4_ (0.6 M), ammonium molybdate (4 mM) and sodium phosphate (28 mM). Samples were incubated at 85oC for 30 min, readings were taken on a spectrophotometer using ascorbic acid as standard [20].

#### Reducing power

Sample (1 ml) was mixed with 2.5 ml potassium ferricyanide (1%) and incubated at 50 °C for 15 min. Supernatant (2.5 ml) was mixed with 0.5 ml ferric chloride (0.1% in distilled water). Absorbance was measured on a double beam spectrophotometer at 700 nm [21].

#### Antioxidant activity in linoleic acid system (ALA)

Antioxidant activity was measured by the described method of Osawa and Namiki [22].

#### Fatty acid profile

Fatty acid profile of UHT-treated tea whitener, milk and dairy drinks samples was determined on a GC-MS (7890-B, Agilent Technologies). Extracted fat 0.5 mg was taken in a test tube, reacted with methanolic hydrogen chloride and put in the heating block for 1 h. Extraction was performed by n-hexane, dried on sodium sulfate and 1 μL was injected through front auto liquid sampler and injected into fused silica capillary column (SP 2560; 100 m, film thickness 25um). Temperature of the inlet and detector were set at 200 and 250 °C, with a split ration of 1:50 using helium, hydrogen and oxygen at the rate of 1 ml/ min, 4 ml/ min and 40 ml/ min, respectively. Quantification was done by FAME-37 standards [23].

#### Use of accelerated oxidation in UHT-treated products

For the assessment of oxidative stability of UHT-treated products, Professional Rancimat (Model 892) was used. For the extraction of fat from samples, protocol of AOAC was used [24]. Briefly, 2.5 g fat was taken in reaction vessels of Rancimat, a steady of dried oxygen was introduced at the flow rate of 20 l/hr. using Synlab software. Induction period was calculated from the breakpoint in the curve [25].

### Statistical analysis

This experiment was conducted in a CRD. Data were analyzed by two-way variance technique and results were express as Mean ± SD. For the determination of significant difference DMR Test was performed on a SAS 9.1 software keeping confidence interval at 95%. *p* < 0.05 was taken to indicate statistical significance.

## Results

Table 1 describes the chemical composition of UHT treated tea-whitener, milk and dairy drink. UHT-treated tea whitener showed significantly higher fat content, no significant difference was recorded in fat content of UHT treated milk and dairy drink. In UHT treated tea-whitener, fat content was toned at 6% level while, in UHT treated milk and dairy drink, fat content was set at 3.5%. At zero day, TAC of UHT treated tea-whitener, milk and dairy drink samples were 45.2, 43.5 and 40.2%, respectively (Table 2). After 45 days of storage, TAC of UHT-treated tea whitener, milk and dairy drink were 41.8, 35.9 and 22.4%, respectively. After 90 days of storage, TAC of UHT-treated tea whitener, milk and dairy drink were 37.2, 27.7 and 15.4%, respectively. At zero day, reducing power of UHT treated tea- whitener, milk and dairy drink were 6.82, 6.22 and 5.29%, respectively. However, it was strongly influenced by the temperature and storage length. After 45 days, reducing power of UHT-treated tea whitener, milk and dairy drink was 5.77, 4.59 and 1.17, respectively and it decreased to 0.24 when UHT-treated products were stored for 90 days. Antioxidant activity in linoleic acid of UHT-treated samples is shown in Table 2. At zero-day, ALA of UHT treated tea-whitener, milk and dairy drink was 10.6%, 9.88 and 8.43%, respectively and decreased with the rise of storage duration. After 45 days of storage, the loss of ALA in UHT treated-tea whitener, milk and dairy drink were 20.3, 39.4 and 50.2%, respectively. After 90 days of storage, the loss of ALA in UHT-treated tea whitener, milk and dairy drink were 51.1, 65.8 and 88.9%, respectively. At zero days, DPPH free radical scavenging activity of UHT treated tea-whitener, milk and dairy drink 25.6, 23.6 and 21.8%, respectively. Storage duration significantly affected the DPPH free radical scavenging activity. After 45 days of storage, the lowest DPPH free radical scavenging activity (7.6%) was recorded in UHT-treated tea-whitener. At zero-day, the contents of vitamin A and E in UHT-treated milk were 0.46 μg/100 g and 0.63 mg/100 g, respectively (Table 3). UHT treated tea-whitener had the lowest concentration of vitamin A and E. After 90 days of storage of UHT-treated tea whitener, milk and dairy drink, the loss of vitamin A was 47.8, 100 and 100%, respectively. After 45 days of storage, the loss of vitamin E in UHT-treated tea whitener, milk and dairy drink was 19.1, 82.5 and 100%, accordingly. After 90 days of storage of UHT-treated tea whitener, milk and dairy drink, the loss of vitamin E was observed 69.8, 100 and 100%, respectively. At zero-day, amount of short, medium and unsaturated fatty acids in UHT-treated milk were 10.54, 59.71 and 27.44%, respectively. After 90 days of storage, amount of medium and unsaturated fatty acids in UHT-treated tea whitener, milk and dairy drink were 54.8 and 25.62%, respectively. After 45 days of storage of UHT-treated tea whitener, the loss of medium and unsaturated fatty acid was 1.6 and 0.99%, respectively. After 90 days of storage of UHT-treated tea whitener, the loss of medium and unsaturated fatty acid was 8.2 and 6.6%, respectively. After 45 days of storage of UHT-treated milk, the loss of short, medium and unsaturated fatty acid was 7%, 7.1 and 5.8%, respectively. After 90 days of storage of UHT-treated milk, the loss of short, medium and unsaturated fatty acid was 8.53, 13.51 and 11.88%, respectively. Samples of UHT treated dairy drink underwent sever oxidation after 45 and 90 days of storage and the loss of C_18:1_ was 14.5 and 32.8%, respectively. At zero-day, induction period of UHT treated tea-whitener, milk and dairy drink were 15.67, 9.74 and 7.27 h (Table 4). After 45 days of storage period of UHT treated tea-whitener, milk and dairy drink, the induction period was 10.35, 8.61 and 5.19 h. After 90 days of storage, induction period of UHT treated tea-whitener was 3.06 h greater than UHT-treated milk.

**Table 1:** Effect of storage duration on chemical composition of UHT-treated tea whitener, milk and dairy drink

UHT-treated Dairy Product	Storage Days	Fat (%)	Protein (%)	Lactose (%)	pH
Tea Whitener	0 45 90	6.00 ± 0.03^a^ 5.94 ± 0.05^a^ 5.90 ± 0.02^a^	3.28 ± 0.07^a^ 3.27 ± 0.04^a^ 3.15 ± 0.09^b^	4.68 ± 0.13^a^ 4.65 ± 0.10^a^ 4.41 ± 0.12^c^	6.65 ± 0.08^a^ 6.64 ± 0.02^a^ 6.52 ± 0.01^a^
Milk	0 45 90	3.50 ± 0.03^a^ 3.42 ± 0.01^a^ 3.35 ± 0.04^a^	3.28 ± 0.07^a^ 3.25 ± 0.03^a^ 3.18 ± 0.01^b^	4.68 ± 0.13^a^ 4.64 ± 0.06^a^ 4.55 ± 0.14^b^	6.65 ± 0.08^a^ 6.61 ± 0.07^a^ 6.54 ± 0.03^b^
Dairy Drink	0 45 90	3.50 ± 0.03^a^ 3.36 ± 0.06^a^ 3.34 ± 0.08^a^	3.28 ± 0.07^a^ 3.24 ± 0.05^a^ 3.09 ± 0.02^b^	4.68 ± 0.13^a^ 4.58 ± ±0.09^b^ 4.57 ± 0.04^b^	6.65 ± 0.08^a^ 6.55 ± 0.03^b^ 6.57 ± 0.0^b^

If means are expressed by a non-uniform letter in a column, these are statistically significant at 95% confidence interval (*p* < 0.05)

**Table 2:** Antioxidant capacity of UHT-treated products at different storage intervals

UHT-treated Dairy Product	Storage Days	TAC (%)	ALA (%)	RP	DPPH (%)
Tea Whitener	0 45 90	45.21 ± 0.41^a^ 41.84 ± 0.29^b^ 37.29 ± 0.55^c^	10.60 ± 0.09^a^ 8.44 ± 0.13^b^ 5.19 ± 0.15^d^	6.82 ± 0.16^a^ 5.77 ± 0.14^b^ 3.69 ± 0.18^d^	25.63 ± 0.24^a^ 21.24 ± 0.19^b^ 18.77 ± 0.15^c^
Milk	0 45 90	43.53 ± 0.41^a^ 35.92 ± 0.88^d^ 27.71 ± 0.91^e^	9.88 ± 0.09^a^ 6.42 ± 0.11^c^ 3.62 ± 0.21^d^	6.11 ± 0.16^a^ 4.59 ± 0.27^c^ 1.91 ± 0.07^e^	23.37 ± 0.24^a^ 16.11 ± 0.13^d^ 11.19 ± 0.49^e^
Dairy Drink	0 45 90	40.24 ± 0.41^a^ 22.40 ± 0.39^f^ 15.44 ± 0.69^g^	8.43 ± 0.09^a^ 5.27 ± 0.15^e^ 1.17 ± 0.09^f^	5.29 ± 0.16^a^ 1.17 ± 0.08^f^ 0.24 ± 0.03^g^	21.85 ± 0.24^a^ 7.57 ± 0.36^f^ 2.14 ± 0.64^g^

If means are expressed by a non-uniform letter in a column, these are statistically significant at 95% confidence interval (*p* < 0.05)

TAC: Total antioxidant capacity; ALA: Activity in linoleic acid; RP: Reducing power

**Table 3:** Vitamin A and E content of UHT-treated tea whitener, milk and dairy drink at different stages of storage

UHT-treated Dairy Product	Storage Days	Vitamin A (μg/100 g)	α-Tocopherol (mg/100 g)
Tea Whitener	0 45 90	0.08 ± 0.02^c^ 0.05 ± 0.04^d^ 0.02 ± 0.01^e^	0.13 ± 0.06^c^ 0.15 ± 0.09^c^ 0.01 ± 0.03^d^
Milk	0 45 90	0.46 ± 0.02^a^ 0.23 ± 0.06 ^b^ 0.09 ± 0.01^c^	0.63 ± 0.06^a^ 0.29 ± 0.02^b^ 0.17 ± 0.01^c^
Dairy Drink	0 45 90	0.11 ± 0.02^c^ ND ND	0.13 ± 0.06^c^ ND ND

If means are expressed by a non-uniform letter in a column, these are statistically significant at 95% confidence interval (*p* < 0.05)

ND: Not Detected

**Table 4:** Induction period and peroxide value of UHT-treated tea whitener, milk and dairy drink at different storage intervals

UHT-treated Dairy Product	Storage Days	Induction Period (Hrs)	PV (MeqO_2_/kg)
Tea Whitener	0 45 90	15.67 ± 0.32^a^ 14.35 ± 0.21^b^ 12.55 ± 0.13^c^	0.22 ± 0.02^g^ 0.48 ± 0.04^f^ 1.71 ± 0.08^d^
Milk	0 45 90	9.74 ± 0.11^d^ 8.61 ± 0.05^e^ 6.55 ± 0.09^g^	0.25 ± 0.02^g^ 1.19 ± 0.12^e^ 2.48 ± 0.19^c^
Dairy Drink	0 45 90	7.27 ± 0.13^f^ 5.19 ± 0.16^h^ 1.84 ± 0.03^i^	0.45 ± 0.02^g^ 3.27 ± 0.16^b^ 5.44 ± 0.13^a^

If means are expressed by a dissimilar letter in a column, it shows non-significant effect at 95% confidence interval (*p* > 0.05)

## Discussion

### Effect of storage on chemical composition of UHT-treated products

Tea whitener was formulated by blending 50% concentrated and 50% toned milk, total solids, protein, fat, ash, acidity and pH were 11.5, 2.2, 1.6, 0.79, 0.2% and 6.3, respectively [26]. Storage effect on fat and protein content of UHT treated tea-whitener, milk and dairy drink revealed a non-significant effect till 90 days of storage. After 90 days, lowest lactose content was found in UHT treated tea whitener followed by UHT milk and dairy drink. Tea-whitener had 5% sucrose, this could be the reason for lower lactose content. Effect of storage on pH of UHT treated tea-whitener, milk and dairy drink was non-significant up to 45 days of storage. After 90 days of storage, fat, protein, lactose contents and pH decreased [3, 27, 28]. pH of UHT treated milk decreased during the long-term storage [29].

### Total antioxidant capacity

Antioxidant systems of milk can be broadly classified into two categories, fat-soluble antioxidant system and the water-soluble antioxidant system. Some of the antioxidants in milk are derived from feed, dairy products containing one or more non-dairy ingredients may have lower antioxidant activity [30]. Lipid oxidation is a serious problem of UHT-treated milk in developing countries. Several factors lead to the development and acceleration of lipid oxidation in UHT treated milk [31]. Total antioxidant capacity (TAC) measures the capability of a substrate to counter with free radicals [32]. In current investigation, TAC was used as a pointer of antioxidant capacity. It was found that TAC of UHT treated tea-whitener, milk and dairy drinks was influenced by the storage time. However, the decline in TAC was more in UHT treated milk and dairy drink samples. Changes in TAC of UHT-treated tea whitener, milk and dairy drink during the storage are not previously studied. Khan et al. [18] reported that antioxidant capacity of pasteurized, boiled, cow and buffalo milk decreased during the duration of 6 days. Peroxide value of UHT treated milk was intensely correlated with TAC. The determination intervals showing higher TAC showed lower peroxide value (R^2^ = 0.9982). Antioxidant activity of milk is largely due to casein, whey, vitamin E, A, C, selenium zinc and enzyme systems. During the storage period of 90 days concentration of fat, protein, α-tocopherol and vitamin A decreased that led to lower TAC in all types of UHT treated products investigated.

### Reducing power

In food system, free radicals cause impulsive oxidation and yield objectionable biochemical compounds which lead to the development of rancidity. Free radicals have also been connected with the development of several diseases in biological systems [33]. Roy and Deepak [34] recorded that milk oligosaccharides had antioxidant activity.

### Antioxidant activity in linoleic acid (ALA)

Balakrishnan and Agrawal [35] recorded variation in antioxidant activity of fermented milk at different stages of processing and storage, it was observed that fermented milk had higher antioxidant activity than non-fermented milk, storage period significantly affected the antioxidant capacity.

### 2, 2-Diphenyl-1-picrylhydrazyle (DPPH) radical scavenging activity

Among the organic radicals, DPPH is widely used for the testing of antioxidant activity of antioxidant molecules [36]. DPPH free radical scavenging assay was used in several milk and dairy fat related studies [17, 37]. Citta et al. [38] reported that DPPH free radical scavenge activity of yoghurt decreased during the storage. Smet et al. [39] used DPPH free radical scavenging activity as an indicator of antioxidant activity in milk. Antioxidant properties of milk and dairy products is strongly correlated with handling,, processing, distribution, storage length and conditions, dairy products formulated one or more non-dairy ingredients may have lower antioxidant properties than pure milk and dairy products [40].

### Vitamin A and tocopherol content in UHT-treated products

UHT-treated products are stored at room temperature for a longer period of time. In storage phase, several chemical changes take place. Rancidity or auto-oxidation is regarded as one of most significant cause of spoilage of UHT treated milk. During storage, lipid oxidation leads to lower the nutritional and sensory perspectives of UHT treated milk and loss of essential fatty acids and vitamins [41]. The effect of storage temperature on physico-chemical characteristics of UHT-treated milk was studied in previous investigations, however, the impact of storage length on vitamin A and vitamin E is not previously studied. In current investigation, the content vitamin A and E were determined because of their antioxidant activities [42, 43]. Vitamin E is the most important lipid-soluble antioxidant, present in milk fat globule membrane and prevent photo-oxidation [44]. For the preparation of wide range of fats, edible oil processors perform partially hydrogenation, this process is performed in the presence of Nickel catalyst at more than 200 °C, for many hours. Severe processing conditions in partial hydrogenation, post hydrogenation refining and deodorization stages, palm oil is exposed to high temperature (> 200 °C) for many hours and this sever heat treatment almost eliminates the naturally occurring vitamins [45]. Naturally occurring tocopherols and other antioxidant substances were destroyed in batch deodorization [46]. For the manufacturing of anhydrous milk fat, a heat treatment is applied which decreases the concentration of fat-soluble vitamins. Concentrations of vitamin A and E was significantly affected by the length of storage period. After 45 days of storage, UHT treated tea-whitener, milk and dairy drink, loss of vitamin A was 23.9, 60.8 and 100%, respectively. An investigation on UHT-treated milk showed that, after 14 days of storage, loss of vitamin A and E was 33 and 11% [47]. Saffert et al. [48] studied the effect of package light transmittance on vitamin A content of UHT-treated milk and loss in vitamin A was noted during the storage.

### Effect of storage duration on fatty acids profile of UHT-treated products

Milk fat contains two different types of fatty acids and these can be broadly categorized into saturated and unsaturated fatty acids. About 70% are saturated and remaining 30% are recognized as unsaturated fatty acids. Milk fat also contains about 10–12% short-chain fatty acids. In nature, milk fat is one the largest source of short-chain fatty acids. These are important for typical flavor of milk and dairy products and medium-chain fatty acids are important for functional properties of milk and other dairy products [17]. From oxidative stability view point, unsaturated fatty acids are important [49]. Lipid oxidation is a recognized as one of the most noticeable reason for the spoilage of UHT-treated milk [50]. In lipid oxidation, fatty acids are broken down to oxidation products which leads to the development of rancid flavor in dairy products [37]. The changes in fatty acid composition are usually used to find out the oxidative stability of fat [51]. Changes in fatty acids profile of UHT-treated milk are mentioned in Table 5. Nadeem and Ullah [37] studied the impact of high storage temperature on fatty acid profile of milk fat and found short, medium and unsaturated fatty acids decreased. Rate of lipid oxidation is dependent upon certain catalysts such as temperature, light and metals [52]. Rate of lipid oxidation is mainly influenced by the fatty acids profile and therefore, partial hydrogenation of liquid oils is performed to increase their oxidative stability [31]. Partial hydrogenation of oils and fats decreases the degree of unsaturation and increases the oxidative stability [53]. Tea whitener was formulated from partially hydrogenated palm oil (iodine value 42 cg/100 g) which could be reason for lower extent of lipid oxidation it tea whitener as compared to UHT milk and [54]. Fatty acids profile of milk fat significantly changed after 180 days of storage [17, 55]. For the assessment of oxidative stability, changes in the fatty acid profile of milk fat was used as a marker [56]. In current investigation, the loss of unsaturated fatty acids was strongly connected with the peroxide value and assessment intervals showing higher losses of fatty acids had higher peroxide value. Lipid oxidation leads to the development of oxidized flavor that limit the shelf life and sensory characteristics of milk [57–59]. Chemcailly, milk fat is complex as compared to vegetable oils and fats, triglyceride profile of milk fat is considerably different from vegetable oils and fts, a detailed investigation on triglyceride profile of immitant dairy products should be performed.

**Table 5:** Fatty acids profile of UHT-treated products under different storage conditions

Fatty acid	UHT-treated Dairy Product								
	Tea Whitener			Milk			Dairy Drink		
	0-Day	45-Days	90-Days	0-Day	45-Days	90-Days	0-Day	45-Days	90-Days
C_4:0_	ND	ND	ND	3.41 ± 0.28^a^	3.32 ± 0.05^b^	3.17 ± 0.05^c^	2.65 ± 0.12^e^	2.31 ± 0.15^d^	2.05 ± 0.12^e^
C_6:0_	ND	ND	ND	2.45 ± 0.29^a^	2.19 ± 0.11^c^	1.94 ± 0.07^d^	1.14 ± 0.03^e^	1.10 ± 0.09^e^	1.03 ± 0.03^e^
C_8:0_	ND	ND	ND	1.44 ± 0.20^a^	1.22 ± 0.05^c^	1.89 ± 0.06^d^	0.62 ± 0.04^e^	0.50 ± 0.02^e^	0.42 ± 0.04^e^
C_10:0_	ND	ND	ND	3.24 ± 0.18^a^	3.07 ± 0.03^c^	2.64 ± 0.16^d^	1.32 ± 0.13^e^	1.25 ± 0.07^e^	1.12 ± 0.13^e^
C_12:0_	0.29 ± 0.11^a^	0.26 ± 0.13^a^	0.19 ± 0.16^b^	3.66 ± 0.11^a^	3.35 ± 0.19^c^	2.88 ± 0.14^d^	1.59 ± 0.02^e^	1.40 ± 0.08^e^	1.29 ± 0.02^e^
C_14:0_	1.01 ± 0.55^a^	0.99 ± 0.42^a^	0.76 ± 0.26^b^	11.97 ± 0.55^a^	10.42 ± 0.36^c^	9.33 ± 0.22^d^	7.64 ± 0.16^e^	7.44 ± 0.31^e^	6.74 ± 0.16^e^
C_16:0_	42.5 ± 1.17^a^	40.2 ± 0.38^a^	37.87 ± 0.33^b^	29.97 ± 1.17^a^	28.33 ± 0.41^c^	27.14 ± 0.43^d^	25.73 ± 0.49^e^	25.51 ± 0.63^e^	24.73 ± 0.49^e^
C_18:0_	3.8 ± 0.99^a^	3.01 ± 0.27^a^	2.89 ± 0.21^b^	14.11 ± 0.99^a^	13.39 ± 0.53^c^	12.29 ± 0.18^d^	10.24 ± 0.37^e^	10.18 ± 0.40^e^	9.24 ± 0.37^e^
C_18:1_	25.8 ± 1.24^a^	25.11 ± 0.73^a^	24.89 ± 0.47^b^	25.61 ± 1.24^a^	24.02 ± 0.31^c^	23.37 ± 0.45^d^	18.43 ± 0.51^e^	18.27 ± 0.22^e^	17.43 ± 0.51^e^
C_18:1[*Trans*]_	18.5 ± 0.79^a^	18.4 ± 0.96^a^	18.1 ± 0.43^a^	ND	ND	ND	ND	ND	ND
C_18:2_	9.52 ± 0.12^a^	8.89 ± 0.03^a^	7.34 ± 0.02^b^	1.37 ± 0.12^c^	1.18 ± 0.02^d^	0.76 ± 0.04^e^	ND	ND	ND
C_18:3_	0.29 ± 0.03^b^	0.21 ± 0.01^c^	0.13 ± 0.04^d^	0.46 ± 0.03^a^	0.25 ± 0.01^b^	0.05 ± 0.01^e^	ND	ND	ND

If means are expressed by a non-uniform letter in a row, these are statistically significant at 95% confidence interval (*p* < 0.05)

ND: Not Detected

### Induction period and peroxide value of UHT-treated products

Induction period is used for the determination of oxidative stability of oils and fats and this technique is recommended by the American Oil Chemists Society. It is used for the determination of oxidative stability of wide range of food products and depending upon the oil/content, the sample preparation is accordingly performed. However, this method is not previously used for the determination of oxidative stability of thermally treated milk. Most of the time, this technique is used to anticipate the shelf life of vegetable fats and oils. We used this method for the determination of oxidative stability of butter oil [37]. In current investigation, induction period of UHT treated milk was determined on a Rancimat at 120 °C with 20-l O_2_/hr. For the determination of induction period of UHT treated milk, the fat was extracted from the milk and 2.5 g sample was used for measurement of induction period on a Rancimat using lab StabNet 1.1 software. Partially hydrogenated fats have better oxidative stability, therefore, induction period of UHT treated tea-whitener was greater than UHT milk and dairy drink. Induction period of UHT-treated milk was affected by the storage period. Strong correlations were observed between induction period and peroxide value. UHT treated product having higher induction period had lower peroxide value and vice versa. Use of induction period for the measurement of oxidative stability of milk is studied in a limited way. Ajmal et al. [60] studied the induction period of UHT treated milk stored for 90 days, it was found that induction period decreased with the progression of storage length. For the determination of oxidative stability of low melting point fractions of buffalo butter oil, induction period was used as a key parameter [40]. Induction period was used for the determination of oxidative stability of whey butter, blend of butter oil and mango kernel oil, blend of butter oil with palm oil etc. [37, 61]. Peroxide value of UHT-treated samples increased with the advancement of storage duration and UHT-treated milk and dairy drink revealed higher peroxide value than UHT-treated tea whitener.

## Conclusion

The results of fatty acid composition of UHT-treated whitener and dairy drink showed that former was formulated from partially hydrogenated fat and latter had anhydrous milk fat, as a source of fat. Tea-whitener had more than 18% harmful *trans* fatty with lower antioxidant properties. Peroxide value and induction period indicated that oxidized fat was used in the formulation of dairy drink. The results of this investigation indicated that regionally developed immitant dairy products should be avoided and milk should be preferred. Further, developing countries should make rules and regulations about immitant dairy products and ensure strict compliance to safeguard the health of large number consumers using these version of dairy products.

## Abbreviations

ALA: Antioxidant Activity in Linoleic Acid
DPPH: 2, 2-Diphenyl-1-picrylhydrazyle
TAC: Total Antioxidant Capacity
UHT: Ultra High Temperature Treatment
